# The "Effort Syndrome" in Relation to Blood Quality

**Published:** 1918-07-06

**Authors:** 


### ON BREATHLESSNESS IN CARDIAC DISEASE.

### The "Effort Syndrome" in Relation to Blood Quality.

BREATHLESSNESS in cardiac valvular disease, and more particularly in mitral disease, is a familiar symptom. It varies widely in degree, and even to some extent in character or form. At one extreme are cases in which the breathlessness is present even when the patient is at rest in bed. Here it is commonly associated with cyanosis, and the patient is compelled for the sake of more efficient inspiratory movement to adopt a sitting posture. Again, there are cases in which the Cheyne-Stokes rhythm is established. The other end of the scale is occupied by cases where the dyspnœa is provoked only on exertion of greater or less severity. Of recent years this symptom has attracted a good deal of attention, and special emphasis has been placed upon it as a graduated index by which can be estimated the reserve power of the heart muscle. Hence it is offered as a valuable guide to prognosis, and this is perhaps generally accepted as an established clinical position. When the clinicians are asked to explain the mechanism by which the breathlessness is brought about they do not always find the task an easy one, and it may be admitted that the explanation is not to them of the first order. Their immediate business is to recognise the symptom and to read it as a guide to the treatment of the patient. With physiological or pathological machinery they are not so directly concerned. Yet the clinical significance of symptoms and the method of their production cannot wisely be divorced from each other, and time and again physiological truths have been found to have a close application to medical treatment. Not that the teaching has always been one-sided. Far from it indeed. Perhaps it would be safe to say that fully as much light has passed from clinical medicine to physiology as in the opposite direction, and certainly the highly desirable thing is to keep the two in close relation the one to the other.

A common phrase used by the physician in reference to the existence of dyspnœa in heart disease is "embarrassment of the lungs." The term has a sonorous quality, though it is somewhat wanting in precision. Yet it is not wide of the mark in the more extreme cases, as, for example, in those where the breathlessness takes the form of orthopnœa. Here, other considerations apart, the presence of cyanosis announces a deficient oxygenation of the blood, and the physical signs of pulmonary œdema and of passive congestion explain how this deficient oxygenation is produced. The difficulty thus appears to be a mechanical one brought about by an overfull condition of the pulmonary circulation, and this in its turn is established either by deficient propulsive force in the right ventricle or by a hindered drainage of the lungs due to an obstructed flow at the mitral orifice. There is a further clinical witness to the accuracy of this conclusion—namely, the fact that it is in cases of this order that the inhalation of oxygen produces benefit. Such a procedure increases the opportunity of oxygen-entrance into the blood, and hence tends to neutralise the deficiency caused by the abnormal state of the circulation in the lungs. Probably, however, there is another influence at work—namely, the influence of oxygen on the viscosity of the blood. Some years ago the late Sir Lauder Brunton described how in endeavouring to bleed a patient he failed to get a satisfactory flow of blood until the patient was made to breathe an atmosphere of oxygen, when almost at once a free blood-flow was established. Presumably the oxygen lessened viscosity, and the desired result followed. In any event, the experience was a piece of practical clinical information of which it is well that due note be taken.

But, whatever may be true of the more severe cases, it is certain that the mechanical explanation will not apply to instances in which pulmonary œdema and congestion do not exist. Here there is no cyanosis, and the inhalation of oxygen has little or no effect. Hence to speak of "embarrassment of the lungs" is to use a form of words without knowledge. The problem has recently been extended, and in some measure elucidated, by an investigation of the cases of soldiers who, in consequence of breathlessness, palpitation, præcordial pain, fainting attacks, etc., on exertion, have been found unfit for full military duty. Very naturally the problem was placed by the Medical Research Committee in the capable hands of Dr. Thomas Lewis, whose researches on various cardiac problems are well known to the profession. Putting aside cases of gross organic disease, Dr. Lewis took a large number of cases, in all of which the symptoms named above were provoked by exertion, and to this group of symptoms he proposes to apply the term "effort syndrome." The phrase is a happy one, for it indicates the facts without any speculative suggestion as to their exact nature. The cases so observed promptly fell into two groups. In one there were physical signs indicating the existence of organic valvular disease. In the other the clinical examination gave no such indication. There thus appeared a *prima facie* case that the valvular flow was not the cause of the breathlessness. In both groups the symptoms were of the same order, and in both they were excited by the same influence. Yet in the one group there were valvular defects and in the other there were no such defects, and, it may be added, the degree of the symptoms and the measure of physical debility were not greater in the valvular than in the non-valvular cases. In these circumstances clearly the valvular flaw could not be held responsible for the symptoms. A series of analytical inquiries as to the personal history helped to the further establishment of this conclusion, for these inquiries showed no higher proportion of influences likely to be prejudicial to cardiac efficiency in the one group than in the other, with the exception of rheumatic fever, which naturally preponderated in the valvular group.

**On Breathlessness in Cardiac Disease—(*continued*).**

In this way the problem took on a new aspect. Valvular flaws—understanding by this the slighter forms that had not been so decided as to exclude the men from the Army or to cause them to break down during training—plainly were not the explanation of the "effort syndrome," for the syndrome was equally present in men in whom no such flaw existed, and who were free from physical signs capable of interpretation as indicating a disturbed pulmonary circulation. Yet some explanation must exist, and, for the reasons given above, the probability is that one and the same explanation applies to both groups of cases. The suggestion now advanced is that the breathlessness is due to an abnormality in certain of the blood-salts. The gaseous changes which the blood, as a consequence of tissue and pulmonary respiration, is constantly undergoing mean a recurring alteration in the measure of its alkalinity, as the fluid constantly receives and discharges both acid and alkaline substances. These modifications would produce considerable and more or less sudden disturbances were it not for the presence in the blood of certain salts which, as it were, tie a considerable proportion of the carbonic acid to themselves and so prevent an excess of the acid being free in the blood plasma. Suppose a deficiency of these salts, there will be an excess of free acid, a condition which for well-established physiological reasons must produce dyspnœa. To the salts which discharge this function of permitting the gaseous interchanges to take place without violent oscillations in the reaction of the blood is given the name "buffer salts," and dyspnœa in such cases as are here contemplated is presented as a consequence of deficiency in these "buffer salts." There are certain chemical investigations advanced in the support of this doctrine, and the whole story may be read in a special report issued by the Medical Research Committee. The matter is one of great interest alike to the physiologist and to the physician.

Since the issue of the report a challenge to the originality, and even to the accuracy, of the main conclusion has appeared. This is advanced by Professor Benjamin Moore in the *British Medical Journal* of June 29. Professor Moore claims that some years ago himself and his colleagues at the University of Liverpool discovered and described the protective action which is now placed to the credit of certain of the inorganic blood-salts. Further, his contention is that such protective action is not exercised by these salts, but by the "amphoteric proteins." He adds a piquant comment. The communication made by himself and his colleagues received little notice in the country of its birth. But the "reactivity of the serum," published in the *Proceedings of the Royal Society*, did not escape attention in Germany, where the term "buffer-salt effect" was coined. Having acquired this dignity the story comes back to the country of its origin, where originally there were none to do it reverence. We may expect to hear more on the matter, but so far the experience certainly seems not unlike other records of which we have read. An ingenious manipulation of the work of others has sometimes been the foundation of the fame of an individual, and perhaps the process may extend to a nation. Anyhow here is a concrete instance which may add one more influence to the suggestion that our prophets might well receive more honour in their own country.

Professor Moore suggests that in the soldiers tested by Dr. Lewis breathlessness may have been due to neuro-muscular fatigue induced by a physical training for which they are unfitted by nature, and he states that the neuro-muscular and cardiac systems may be "chronically fatigued" by variations in the proportions of organic acids, alkali, and carbonic acid in the blood-content.

